# Low energy availability: history, definition and evidence of its endocrine, metabolic and physiological effects in prospective studies in females and males

**DOI:** 10.1007/s00421-020-04516-0

**Published:** 2020-10-23

**Authors:** José L. Areta, Harry L. Taylor, Karsten Koehler

**Affiliations:** 1 Research Institute for Sport and Exercise Sciences, Liverpool John Moores University, Tom Reilly Building, Byrom St Campus, Liverpool, L3 3AF UK; 2 Department of Sport and Health Sciences, Technical University of Munich, Munich, Germany

**Keywords:** Energy availability, Energy balance, RED-S, Triad, Exercise, Nutrition

## Abstract

Energy availability (EA) is defined as the amount of dietary energy available to sustain physiological function after subtracting the energetic cost of exercise. Insufficient EA due to increased exercise, reduced energy intake, or a combination of both, is a potent disruptor of the endocrine milieu. As such, EA is conceived as a key etiological factor underlying a plethora of physiological dysregulations described in the *female athlete triad*, its male counterpart and the *Relative Energy Deficiency in Sport* models. Originally developed upon female-specific physiological responses, this concept has recently been extended to males, where experimental evidence is limited. The majority of data for all these models are from cross-sectional or observational studies where hypothesized chronic low energy availability (LEA) is linked to physiological maladaptation. However, the body of evidence determining causal effects of LEA on endocrine, and physiological function through prospective studies manipulating EA is comparatively small, with interventions typically lasting ≤ 5 days. Extending laboratory-based findings to the field requires recognition of the strengths and limitations of current knowledge. To aid this, this review will: (1) provide a brief historical overview of the origin of the concept in mammalian ecology through its evolution of algebraic calculations used in humans today, (2) Outline key differences from the ‘energy balance’ concept, (3) summarise and critically evaluate the effects of LEA on tissues/systems for which we now have evidence, namely: hormonal milieu, reproductive system endocrinology, bone metabolism and skeletal muscle; and finally (4) provide perspectives and suggestions for research upon identified knowledge gaps.

## Introduction

Insufficient dietary energy intake can disrupt normal homeostasis in humans, an effect that can be exacerbated by the energetic demands of sports and exercise. The models of the *female athlete triad* (De Souza et al. 2014)*,* its male counterpart (De Souza et al. 2019a, 2019b; Tenforde et al. 2016) and the *relative energy deficiency in sport (RED-S)* (Mountjoy et al. 2018a) provide theoretical frameworks for physiological dysregulations and negative health and performance outcomes triggered by a low energy intake relative to energy expenditure in humans who exercise regularly. Lying at their core is the concept of *energy availability (EA)*, a concept that provides a single numerical value that is thought as the aetiological factor of a broad range of hormonal, metabolic and physiological dysregulations triggered by energy deficiency (De Souza et al. 2014, 2019b; Mountjoy et al. 2014)*.*

The most current description of energy availability (EA) defines it as the difference between energy intake (EI) and exercise energy expenditure (EEE), expressed relative to an individual’s lean body mass (LBM, Table 1), representing the daily amount of energy ‘available’ to sustain all physiological functions outside of exercise (Loucks 2020). Research addressing the effect of different levels of EA on hormonal status of the hypothalamic-pituitary-thyroid axis (Loucks and Heath 1994a), hypothalamic-pituitary-ovarian axis (Loucks and Thuma 2003) and markers of bone resorption and formation (Ihle and Loucks 2004) in young lean sedentary women established a ‘threshold’ of ~ 30 kcal/kg LBM/day below which a disruption of the normal hormonal milieu occurs. Although the existence of specific EA thresholds is debated in females (De Souza et al. 2019c; Lieberman et al. 2018), and unknown in males, it has been shown that short periods of ‘low’ energy availability (LEA, normally considered to be ≤ 30 kcal/kg LBM/day) trigger acute endocrine, metabolic and physiological dysregulations (see Tables 2, 3, 4 and 5) that, when maintained over long periods of time, are believed to result in adverse health and functional outcomes.

**Table 1:** Overview of the evolution of the concept of energy availability through time

	Original concept	1st Algebraic definition	2nd Algebraic definition	3rd Algebraic definition
Algebraic formula	None	EA = (EI − TEEE)/BW	EA = (EI − TEEE)/LBM*	EA = (EI − EEE)/LBM
Taxon of focus	Mammals (experimental work mostly in rodents)	Humans	Humans	Humans
Key new characteristic in concept, definition or formula	• Variation in nutrient availability modulates reproductive behaviour/ readiness in mammals • Both ingested and stored metabolic fuel availability play a role in rodent models • Reductions in carbohydrate and fat availability are necessary for suppression of reproductive function in rodents	• 1st Algebraic definition providing a quantifiable parameter • Total exercise energy expenditure (TEEE) is defined as the gross amount of energy expended during exercise • Relativises EA to body weight (BW) • Focuses on ingested fuel availability (applies to all algebraic definitions)	• Relativises EA to lean body mass	• Exercise Energy Expenditure (EEE) is calculated by subtracting non-exercise energy expenditure (resting metabolic rate + non-exercise waking activity) from TEEE
First described in	Schneider and Wade (1989) Bronson (1985)	Loucks and Callister (1993)	Loucks and Heath (1994a)	Loucks et al. (1998)
EA value example^†^	N/A	40 kcal/kg LBM/day	47 kcal/kg LBM/day	50 kcal/kg LBM/day

The algebraic definitions have been numbered following earliest identification in the literature

*Here and throughout the manuscript we refer to the sum of the active protoplasm as ‘Lean Body Mass’ (LBM) for the sake of uniformity acknowledging small differences with ‘Fat Free Mass’ (FFM), that has also been used in the literature to refer to metabolically active tissues

^†^This example is based on a 75 kg body mass individual with 15% fat mass, exercising for 1 h with a gross energy expenditure of 700 kcal/h; changes of any of these parameters will yield different differences between EA calculations

**Table 2:** Summary of experimental studies investigating the response of key hormones with divergent amounts of energy availability

Study	LEA Induction Method	Population	LEA Duration (days)	EI	EEE	EA	Exercise Mode	Dietary Composition (Pro, CHO, Fat %)	Insulin	Glucose	β-HOB	Leptin	T_3_	GH	IGF-1	Cortisol
Loucks and Heath (1994a)	REI Only	Y, SED (*N* = 7 F)	4^†^	10 and 43	0	10 and 43	N/A	15, 55, 30%	↓ ^§^	NA	NA	NA	↓ ^§^	NA	↓ ^§^	⟷ (24 h $\overline{x}$)
Loucks, Verdun and Heath (1998)	EX Only	Y, SED (N = 9 F)	4^†^	40 and 75	30	13 and 46	Running @ 70% VO_2max_	15, 55, 30%	↓ ^§^	↓ LEA vs NEA (24 h $\overline{x}$)	NA	NA	↓ ^§^	↑ ^§^	↓ ^§^	↑ (24 h $\overline{x}$)
Loucks and Thuma (2003)	REI and EX (3 × 2 Exp. Design)	Y, SED (*N* = 29 F)	5^†^	25, 35, 45 and 60	15	10, 20, 30 and 45	Running @ 70% VO_2max_	15, 57, 28%	↓ in all LEA; and 10 < 30 (24 h $\overline{x}$)	↓ in all LEA; 10 < 20 and 30; 10, 20 and 30 < 45 (24 h $\overline{x}$)	10 > 20 > 30 > 45^§^	↓ in all LEA; and 10 < 30^§^	↓ in all LEA^§^	↑ in EA < 20 (24 h $\overline{x}$)	↓ in EA < 20^§^	↑ in all LEA (24 h $\overline{x}$)
Loucks and Verdun (1998)	REI and EX	Y, SED (*N* = 8 F)	5 (+ 1-day RF)	22 (+ 89 RF day)	15	10 (+ 77 RF day)	Running @ 70% VO_2max_	15, 55, 30%	↓ in LEA, ↑ to baseline by RF^§^	↓ in LEA, ↑ to baseline by RF^§^	↑ in LEA, ↓ to baseline by RF^§^	NA	↓ in LEA, ⟷ after RF^§^	NA	NA	NA
Loucks (2006)	REI and EX	SED (*N* = 19 F; 9 ADOL, 10 ADULT)	5^†^	25 and 60	15	10 and 45	Running @ 70% VO_2max_	15, 57, 28%	↓ in LEA (both groups, 24 h $\overline{x}$)	↓ in LEA (both groups, 24 h $\overline{x}$)	↑ in LEA^§^ (both groups)	↓ in LEA^§^ (both groups)	↓ in LEA^§^ (both groups)	↑ in LEA (both groups) ↑↑ in adults (24 h $\overline{x}$)	↓ in LEA (both groups)	↑ in LEA (both groups; 24 h $\overline{x}$)
Loucks and Callister (1993)	REI only; REI and EX (3 × 2 Exp. Design)	Y, SED (*N* = 46 F)	4	8, 20 and ~ 50*	0 and ~ 21*	8 and 29*	Running @ 70% (HI) or 40% (LI) VO_2max_	15, 55, 30%	NA	NA	NA	NA	↓ in LEA^§^	NA	NA	NA
Loucks and Heath (1994b)	REI and EX (3 × 2 Exp. Design)	Y, SED (*N* = 27 F)	4	40, 49, 53 and 68	~ 30	11, 19, 25 and 40	Running/cycling @ 70% VO_2max_	15, 55, 30%	NA	NA	NA	NA	↓ in LEA < 19^§^	NA	NA	NA
Hilton and Loucks (2000)	REI only; Ex only	Y, SED (*N* = 16 F)	4	10 and 45 (REI) 40 and 75 (Ex)	0 (REI) 30 (Ex)	10 and 45 (REI) 10 and 45 (Ex)	Walking @ 70% VO_2max_	15, 55, 30%	NA	NA	NA	↓ 24-h mean and diurnal amp. (2 × in REI)	NA	NA	NA	NA
Koehler et al (2016b)	REI only; REI and Ex	Y, ACTIVE (*N* = 6 M)	4	16, 30, 40 and 52	0, 15, 0 and 15	16, 16, 40 and 38	60% VO_2Peak_	10–15, 50–55, 30–35%	↓ in all LEA	↓ in all LEA	NA	↓ in all LEA	⟷	NA	⟷	NA
Papageorgiou et al (2017)	REI and EX	Y, ACTIVE (*N* = 22; 11 M, 11 F)	5	30 and 60	15	15 and 45	Running @ 70% VO_2max_	~ 18, 48 and 34%	↓	NA	NA	↓ in F, ⟷ In M	↓	NA	↓	NA
Papageorgiou et al (2018)	REI Only; EX only	Y, ACTIVE (*N* = 10 F)	3	15, 45 and 45	0, 30 and 0	15, 15 and 45	Running @ 70% VO_2max_ (LEA (EX) only)	20, 50 and 30%	⟷	NA	NA	↓ in both LEA conditions	↓ in REI LEA trial only	NA	↓ in both LEA conditions	NA
Murphy and Koehler (2020)	REI only	Y, RT (*N* = 7; 5 M, 2 F)	3	15, 15 and 40	0	15, 15 and 40	5 × 5 Back Squats @ ~ 1 RIR	38, 50, 12% (LEA) 14, 60, 26% (CON)	NA	NA	NA	NA	NA	↑ in LEA (both conditions)	↓ in LEA post-REX (both conditions)	NA
Kojima et al (2020)	REI and EX	Y, ET (*N* = 7 M)	3^†^	38 and 72	19	19 and 53	Running @ 70% VO_2max_	~ 21, ~ 54 ~ 25% (LEA) ~ 16, ~ 57, ~ 27% (CON)	NA	⟷	NA	NA	NA	NA	↓ in LEA	NA

Arrows represent changes in groups with low energy availability (typically lower than 40–45 kcal/kg lean body mass/day). A graphical summary of the contents of the table can be seen in Fig. 3

*ADOL* Adolescents, *ADULT* Adults, *CHO* carbohydrates, *CON* control, *EA* energy availability, *EEE* exercise energy expenditure, *EI* energy intake, *ET* endurance trained, *EX* exercise, *ET* endurance-trained, *F* female, *HI* high intensity, *LEA* low energy availability, *LI* low intensity, *M* male, *NA* not assessed, *NEA* normal energy availability, *PRO* Protein, *REI* restricted energy intake, *RIR* repetitions in reserve, *RF* refeeding, *RT* resistance-trained, *SED* sedentary, *Y* young. EA, EI and EEE are reported as kcal/kg LBM/day. Morning blood values are differentiated with §, in studies with serial blood sampling, which also report 24 h transverse means (24 h $\overline{x}$) in selected parameters. †, Sampling/measurements were performed on the 5th or 6th day where the serial blood sampling was performed during 24 h

*For Loucks and Callister (1993) values of EI, EEE and EA are relative to body mass and not LBM

**Table 3:** Summary of experimental studies investigating the response of hormones of the hypothalamic-pituitary-ovarian axis with divergent amounts of energy availability

Study	LEA Induction Method	Population	LEA Duration (days)	EI	EEE	EA	Exercise Mode	Dietary Composition: (Pro, CHO, Fat %)		LH	E_2_	FSH
Loucks and Heath (1994a, b)	REI Only	Y, SED (*N* = 7 F)	4^†^	10 and 43	0	10 and 43	N/A	15, 55, 30%	↓ pulse freq., ↑ amp. in LEA		⟷^§^	⟷^§^
Loucks, Verdun and Heath (1998)	EX Only	Y, SED (*N* = 9 F)	4^†^	37 and 70	24	13 and 46	Running @ 70% VO_2max_	15, 55, 30%	↓ pulse freq., ↑ amp. in LEA		⟷ ^§^	↑ (24 h $\overline{x}$)
Loucks and Thuma (2003)	REI and EX (3 × 2 Exp. Design)	Y, SED (*N* = 29 F)	5^†^	25, 35, 45 and 60	15	10, 20, 30 and 45	Running @ 70% VO_2max_	15, 57, 28%	↓ pulse freq., ↑ amp in LEA < 30		↓ 10 vs 45 (24 h $\overline{x}$)	⟷ ^§^
Loucks and Verdun (1998)	REI and EX	Y, SED (*N* = 8 F)	5 (+ 1-day RF)	22 (+ 89 RF day)	15	10 (+ 77 RF day)	Running @ 70% VO_2max_	15, 55, 30%	↓ pulse freq., ↑ pulse amp. in LEA Partial restoration of pulsatility with 1-day RF, ⟷ amplitude		⟷ ^§^	NA
Loucks (2006)	REI and EX	SED (*N* = 19 F; 9 ADOL, 10 ADULT)	5^†^	25 and 60	15	10 and 45	Running @ 70% VO_2max_	15%, 57%, 28%	↓ pulse freq. and mean 24 h in LEA in ADOL only		⟷ ^§^	NA

Arrows represent changes in groups with low energy availability (typically lower than 40–45 kcal/kg lean body mass/day). A graphical summary of the contents of the table can be seen in Fig. 3

*ADOL* adolescents, *ADULT* adults, *amp.* amplitude, *CHO* carbohydrates, *CON* control, *EA* energy availability, *EEE* exercise energy expenditure, *EI* energy intake, *ET* endurance Trained, *EX* exercise, *ET* endurance-trained, *F* female, *freq.* frequency, *HI* high intensity, *LEA* low energy availability, *LI* low intensity, *M* male, *NA* not assessed, *NEA* normal energy availability, *Pro* protein, *REI* restricted energy intake, *RIR* repetitions in reserve, *RF* refeeding, *RT* resistance-trained, *SED* sedentary, *Y* young. EA, EI and EEE are reported as kcal/kg LBM/day. Morning blood values are differentiated with §, in studies with serial blood sampling, which also report 24 h transverse means (24 h $\overline{x}$) in selected parameters

^†^Sampling/measurements were performed on the 5th or 6th day where the serial blood sampling was performed during 24 h

**Table 4:** Summary of studies investigating the effects of energy availability on markers of bone metabolism

Study	LEA induction method	Population	LEA duration (days)	EI	EEE	EA	Exercise mode	Dietary composition (Pro, CHO, Fat %)	Bone formation markers	Bone resorption markers
Ihle and Loucks (2004)	REI and EX (3 × 2 Exp. Design)	Y, SED (*N* = 29 F)	5	25, 35, 45 and 60	15	10, 20, 30 and 45	Uphill walking @ 70% VO_2max_	15, 57 and 28%	Linear ↓ in P1CP as EA decreased Curvilinear ↓ in OC, greatest between 30 – 20 LEA treatments	NTx ↑ in 10 LEA condition
Papageorgiou et al (2017)	REI and EX	Y, ACTIVE (*N* = 22; 11 M, 11 F)	5	30 and 60	15	15 and 45	Running @ 70% VO_2max_	~ 18, 48 and 34%	P1NP ↓ in F during LEA, ⟷ in M ⟷ in Sclerostin between conditions in M or F	β-CTX ↑ in F during LEA, ⟷ in M
Papageorgiou et al (2018)	REI Only; EX only	Y, ACTIVE (*N* = 10 F)	3	15, 45 and 45	0, 30 and 0	5, 15 and 45	Running @ 70% VO_2max_ (LEA (EX) only)	20, 50 and 30%	P1NP ↓ in LEA (Diet) compared to CON	⟷ in β-CTX
Murphy and Koehler (2020)	REI only	Y, RT (*N* = 7; 5 M, 2 F)	3	15, 15 and 40	0 (days 1—2) REX on day 3 (EEE NR)	0, 0 and 40	5 × 5 Back Squats @ ~ 1 RIR	38, 50, 12% (LEA) 14, 60, 26% (NEA)	⟷ in P1NP ↑ Sclerostin prior to REX bout, restored post-REX	NA

A graphical summary of the contents of the table can be seen in Fig. 3

Arrows represent changes in groups with low energy availability (typically lower than 40–45 kcal/kg lean body mass/day)

*EA* energy availability, *LEA* low energy availability, *EI* energy intake, *EEE* exercise energy expenditure, *CHO* carbohydrates, *PRO* protein, *M* male, *F* female, *ET* endurance-trained, *NA* not assessed, *NEA* normal energy availability, *REI* reduced energy intake, *REX* resistance exercise, *RT* resistance-trained, *Y* young, *P1CP* carboxy-terminal propeptide of type 1 procollagen, *P1NP* N-terminal propeptide of type 1 procollagen, *NTx* aminoterminal telopeptide of type 1 collagen, *β-CTX* C-terminal telopeptide of type 1 collagen, *OC* Osteocalcin

**Table 5:** Summary of studies investigating the effect of energy availability with a focus on skeletal muscle responses after endurance and resistance-type training

Study	LEA induction method	Population	LEA Duration (days)	EI	EEE	EA	Exercise mode	Dietary Composition (Pro, CHO, Fat %)	MPS	Intracellular Signalling	Gene Expression	Skeletal Muscle Glycogen
Areta et al. (2014)	REI and EX	Y, RT (*N* = 15; 8 M, 7 F)	5	30 and 45*	Different across individuals*	30 and 45	REX*	21, 46, 32% (LEA) 15, 41, 44% (NEA)	↓(M and F)	⟷ p-Akt Ser^473^, p-mTOR Ser^2448^, p-p70 S6K Thr^389^, p-rpS6 Ser^235/236^, p-AMPK Thr^172^, p-4E-BP1 Thr^36/47^ or p-eEF2 Thr^56^	↓SLC7A5 after REX ⟷MuRF-1, Atrogin 1, SLC38A 2	NA
Smiles et al. (2015)	REI and EX	Y, RT (*N* = 15; 8 M, 7 F)	5	30 and 45*	Different across individuals*	30 and 45	REX*	21, 46, 32% (LEA) 15, 41, 44% (NEA)	NA	⟷ in p-38-MAPK Tyr^180/182^, p-p53 Ser^15^, p-eIF2α Ser^51^, p-FOXO1 Thr^24^ or p-FOXO3a ^Ser253^	⟷ in beclin-1, Atg12, Atg4b, GABARAP, LC3b /BNIP3 ⟷ in SIRT1, FOX01 or PGC-1α	NA
Ishibashi et al. (2020)**	REI and EX	Y, ET (*N* = 6 M)	3	~ 37 and ~ 71	~ 20	~ 17 and ~ 51	Running @ 70% VO_2max_	21, 54, 25% (LEA) 16, 57, 27% (CON)	NA	NA	NA	↓
Kojima et al. (2020)	REI and EX	Y, ET (N = 7 M)	3	38 and 72	19	19 and 53	Running @ 70% VO_2max_	~ 21, ~ 54, ~ 25% (LEA) ~ 16, ~ 57, ~ 27% (CON)	NA	NA	NA	↓
Areta et al. (2020)	REI and EX	Y, ET (*N* = 9 M)	~ 0.5	49 and 70	30	19 and 40	Cycling (HIT, glycogen depletion)	11, 16, 73% (LEA) 37, 53, 10% (CON)	NA	⟷ p-AMPK Thr^172^, p-ACC^Ser79^, p-4E-BP1 Thr^36/47^, p-p70 S6K Thr^389^, p-eEF2 Thr^56^, p-Akt Ser^473^, p-eEF2 Thr^56^	↑ p53, ⟷PGC-1α, CS, CYCS, PRKN, NRF2, TFAM, COX4, SIRT1, MFN2, DRP1, SCO2, CPT1, CD36, PDK4	⟷

A graphical summary of the contents of the table can be seen in Fig. 3

Arrows represent changes in groups with low energy availability (typically lower than 40–45 kcal/kg lean body mass/day)

*CHO* carbohydrates, *CON* control, *EA* energy availability, *EI* energy intake, *EEE* exercise energy expenditure, *ET* endurance-trained, *EX* exercise, *HIT* high intensity training, *LEA* low energy availability, *NEA* normal energy availability, *NA* not assessed, *REI* reduced energy intake, *REX* resistance exercise, *RT* resistance-trained, *Y* young

*This energy intake is approximate, the subjects of this study had different individual training regimes of REX. Data for EEE and EI not reported relative to LBM

**Calculated manually from data available hence discrepancy of these values from reported mean EA in the paper

While the long-term physiological effects of LEA are thought to be vast and affect a wide range of tissues and systems such as bone, muscle, endocrine axes and the immune system—amongst others—(De Souza et al. 2014, 2019b; Mountjoy et al. 2014, 2018a), there is limited research establishing a *causal link* between EA and its physiological effects (Tables 2, 3, 4 and 5). Instead, a large part of the literature that forms the basis for the *triad* and *RED-S* models relies on cross-sectional studies in populations at risk of being under chronic LEA, including athletes in sports which emphasize leanness or low body weight (such as weight bearing, weight-categorised or aesthetic sports), athletes with disordered eating, or simply populations with eating disorders (Gibbs et al. 2013; Melin et al. 2015; Staal et al. 2018; Warren 2011), as well as observational studies linking endocrine, metabolic and other parameters with EA (Elliott-Sale et al. 2018; Logue et al. 2018, 2020). In many cases, EA is estimated, which is further complicated by the fact that measuring it in the field is difficult and prone to errors due to limitations in the assessments of energy intake and expenditure (Burke et al. 2018; Heikura et al. 2017). While cross-sectional and field studies are essential for the development of hypotheses, the appropriate methodological approach for establishing a *causal* link between LEA and physiological outcomes is by manipulating LEA under controlled experimental conditions. To date, there is no single body of work summarising the current scientific literature determining the physiological effects EA in controlled settings. Considering the emerging role of LEA as a pivotal concept to guide healthy dietary practices in exercising individuals (De Souza et al. 2019a; Logue et al. 2020; Mountjoy et al. 2018a) we believe that a critical overview of the concept of EA as well as an up-to-date summary of the main scientific findings is much needed.

Therefore, the aim of this review is to provide a critical overview of the concept of energy availability, and of all experimental studies assessing the effect of manipulating energy availability in well-controlled settings, to further our understanding of the strengths and limitations of the concept, short-term LEA studies, and their applicability to the field. To this end, this review will (1) provide an overview on the origin and evolution of the concept of energy availability in humans and its calculation, (2) signpost the strengths and limitations of the concept in relation to energy balance, (3) provide a detailed overview of the main findings of all the clinical studies to date directly addressing the endocrine, metabolic and physiological effects of reduced energy availability in humans in controlled settings, and, (4) provide perspectives and suggestions for future research.

While this review refers to the *triad* and *RED-S* models, its aim is not to analyse, criticise or challenge their validity but to provide an overview of the fundamental concept at their core. For further information on these models, the reader is referred to excellent consensus statements, reviews and scientific debates (De Souza et al. 2014, 2019b; Mountjoy et al. 2014, 2018a, 2018b; Williams et al. 2019).

## The birth and history of energy availability as a concept and its calculation

The current concept and algebraic definition of EA and its application to humans has evolved gradually over time. In its origin, the large incidence of secondary amenorrhea observed in exercising women compared to their non-exercising counterparts brought attention to physiological dysregulations possibly triggered by exercise (Loucks and Horvath 1985), under the suspicion that ‘energy drain’, and not stress of exercise *per se* was the underlying cause (Warren 1980). Further research into this phenomenon developed into the now recognised clinical condition identified as the *female athlete triad*, initially arising from the consensus of the likely coexistence of disordered eating, amenorrhea and low bone mineral density in exercising women (Yeager et al. 1993). It was not until 1994 where the first prospective study linked low energy availability with dysregulation of luteinizing hormone (LH), reporting a resemblance of impaired LH pulsatility in females under conditions of low energy availability to those with hypothalamic amenorrhea (Loucks and Heath 1994b), and it was not until 2007 when low energy availability was incorporated officially as an aetiological factor for the triad (Nattiv et al. 2007; Otis et al. 1997). It was earlier, however, where the concept of energy availability was introduced to human clinical trials research (Loucks and Callister 1993), and even earlier where the concept arose in literature.

*Energy availability in humans and its origin from research assessing reproductive function in mammals* The origin of the concept seems to stem from studies evaluating key parameters of mammalian reproductive success in an ecological context, such as nutrient availability and the ratio between food intake and energy expenditure from thermoregulatory and foraging requirements (Bronson 1985, 1989). Subsequently, laboratory studies directly determined the effect of availability of metabolic fuels on reproductive function in hamsters (Schneider and Wade 1989, 1990a). Schneider and Wade (1989) originally defined ‘*availability of metabolic fuels*’ as fat or carbohydrates available to be oxidised by the cells, regardless of their endogenous (i.e., glycogen or fat) or exogenous (dietary macronutrients) origin. However, none of these studies incorporated energy availability as a measurable parameter. The first use of the concept of energy availability in human trials is observed in a study in relation to induction of low-T_3_ syndrome in women exposed to a short period of low energy availability (Loucks and Callister 1993). This study introduced an algebraic definition of energy availability (EA = (EI − Total EEE)/BM), coined by Professor Loucks (Table 1), into the literature.

This original definition evolved through time (Loucks 2020) with three sequential algebraic formulas which were refined with the aim of better representing the dietary energy available for key tissues and systems (Table 1). In the first definition, EA is expressed as energy intake minus gross exercise energy expenditure reported relative to total body mass (Loucks and Callister 1993). In the second definition, lean body mass is recognised as the relevant tissue pool for EA and therefore EA, expressed as energy intake minus gross exercise energy expenditure, is reported relative to lean body mass (Loucks and Heath 1994b). In the third and most current definition, it is recognised that the value of gross exercise energy expenditure used in the previous definitions includes contribution of resting metabolic rate and non-exercise activity that should be subtracted to provide a net value of exercise energy expenditure (Loucks et al. 1998). Further details of these definitions can be found elsewhere (Loucks 2014, 2020).

We believe that these distinctions, particularly in relation to the 3rd (and newest) algebraic definition, have not been clear or evident to a large number of researchers—ourselves included—and may have resulted in researchers and practitioners using different definitions and calculations of energy availability. Given that the same data-set would yield increasing EA values when using more recent equations (1st < 2nd < 3rd, Table 1), this makes it difficult to compare EA values between different studies and to develop, extrapolate, and apply thresholds for use in practice.

In conclusion, the notion of energy availability in humans is different conceptually from what it was in its inception. In humans it was developed as a simple algebraic definition and subsequently identified as the main etiological factor of the female athlete triad and RED-S and evolved over time (Table 1). Beyond these differences, other parameters that may complicate the comparison of EA between studies are those related to total daily energy expenditure, which will be outlined in the following section.

## Energy availability and energy balance, strengths and limitations of current concepts

The main aim of this section is to highlight differences between the concepts of energy availability and energy balance, critically evaluate the strengths and limitations of each, and provide insights into them that have previously not been stressed in the literature. This will allow for better interpretation of the literature discussed in the following sections, as well as provide a background for the discussion of the current research and future research perspectives (see Sects. “Endocrine, metabolic and physiological effects of low energy availability in humans in controlled settings” and “Perspectives and future research”).

**Fig. 1:**
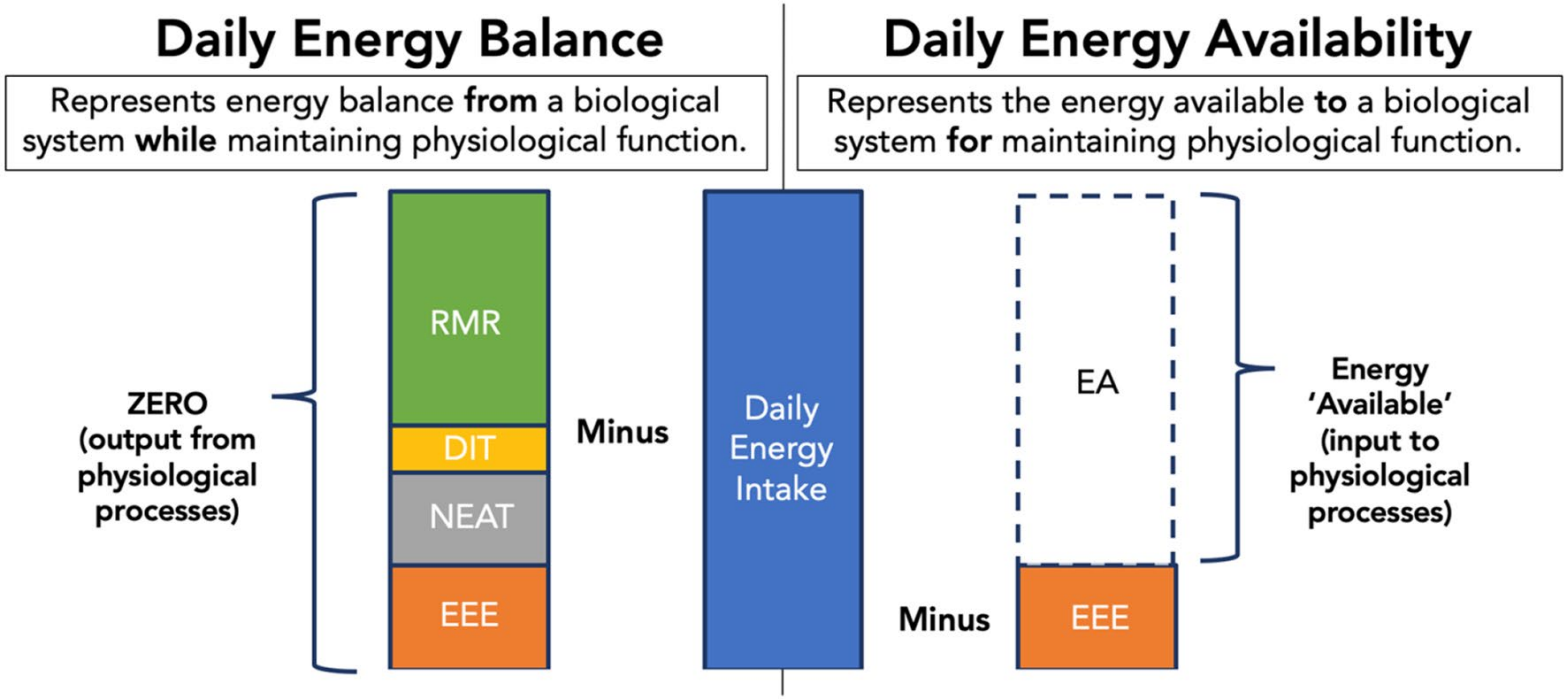
Unit-less illustration of an individual’s daily energy balance and energy availability (EA) when exercising and maintaining an energy balance of zero. Energy intake and exercise energy expenditure (EEE) are accounted for equally in both concepts, but energy balance also accounts for all other components of energy expenditure. The illustration represents parameters of a hypothetical case of an individual performing ~ 1 h of purposeful exercise, with energy expenditure from dietary induced thermogenesis and non-exercise activity thermogenesis that are 10% that of energy intake and 40% of resting metabolic rate, respectively. Cold-induced thermogenesis, has not been incorporated in the figure due to minimal energy expenditure in thermo-neutral conditions, though in cold conditions contribution can be significant. *EA* energy availability, *EEE* exercise energy expenditure, *NEAT* non-exercise activity thermogenesis, *DIT* dietary induced thermogenesis, *RMR* resting metabolic rate

*Similar, but not the same. How are these concepts different?* While the concepts of energy availability and energy balance may appear similar because they both relate energy intake to energy expenditure, their focus is fundamentally different. Energy balance accounts for all components of energy expenditure, while energy availability focuses on exercise energy expenditure. The concept of energy balance relates energy intake to all components of energy expenditure and is typically used in the context of changes in body weight and/or body composition induced by diet and/or exercise interventions. In contrast, the concept of energy availability relates energy intake only to exercise energy expenditure and thereby refers to the amount of energy available to maintain other physiological function outside of exercise (Fig. 1).

Within the concept of energy balance, energy expenditure is typically broken down in its main components: Resting Metabolic Rate (RMR), physical activity expenditure—which is further broken down into exercise energy expenditure (EEE) and Non-Exercise Activity Thermogenesis (NEAT)—dietary-induced thermogenesis (DIT) and Cold-induced Thermogenesis (CIT) (Müller and Bosy-Westphal 2013) (Fig. 1). From a thermodynamic standpoint, it follows that when the difference between energy intake and total energy expenditure is positive, the result is weight gain, whereas weight loss occurs when energy balance is negative. The concept of energy availability, instead, only accounts for EEE, and the simplicity and minimalism of this stands out in Fig. 1. However, the apparent limitation and over-simplification of energy availability may represent the main strength of this concept.

*When balance is out of balance* Achieving an energy balance value of zero does not mean that a healthy metabolic balance has been reached. Metabolism is dynamic and energy expenditure changes as a function of energy balance, a phenomenon frequently referred to as ‘adaptive thermogenesis’ or ‘metabolic adaptation’ (Müller and Bosy-Westphal 2013). As a result, the resulting energy balance is a moving target and—as with most physiological systems—is destined to return to an equilibrium. For example, while individuals entering a negative energy balance first observe weight loss, they experience reductions in most—if not all—components of energy expenditure which results in a decrease in the initial energy deficit (Hall and Kahan 2018; Müller and Bosy-Westphal 2013; Rosenbaum and Leibel 2010). This ‘energy saving’ mechanism is understood as a deeply inbuilt mechanism from our evolutionary legacy to preserve essential tissues and functions during periods of starvation (Rion and Kawecki 2007). This reduction in total energy expenditure is a consequence of reductions in the energy allocated to maintain physiological functions of tissues and organs, which is measurable as a decline in RMR as high as ~ 10–20% (Koehler et al. 2016a; Kosmiski et al. 2014), as well as a reduction in the amount of energy spent being physically active (Müller and Bosy-Westphal 2013; Rosenbaum and Leibel 2010; Rosenbaum et al. 2003). To provide an analogy appropriate to the modern world, these adaptive reductions in various components of energy expenditure resemble a mobile phone switching automatically to a ‘power saving mode’ when the battery is running low. It is reasonable to hypothesize that this newly ‘adapted’ state is associated with a sub-optimal functioning of at least some endocrine and metabolic systems due to down-regulation of their function to preserve energy for crucial systems (see Sect. “Endocrine, metabolic and physiological effects of low energy availability in humans in controlled settings”). More importantly, the result of these adaptations is that energy balance and consequently weight stability is achieved at a lower set-point, thereby representing an apparent state of homeostasis that masks the real lack of energy available for optimal physiological functions.

*Energy availability and adaptive thermogenesis* As energy availability only accounts for intake and EEE and is therefore defined as an input into all physiological systems (Loucks 2004, 2007a, 2007b,^ 2013^,^ 2014^; Loucks et al. 2011), its numerical value is by definition independent of all the other (dynamic) components of energy expenditure outside of exercise, and therefore unaffected by adaptive thermogenesis/energy conservation. This concept implies that optimal physiological function relies on an ‘adequate’ amount of energy availability and allows us to quantify the adequacy of energy available for metabolic processes independent of metabolic adaptation and body weight changes.

*Limitations of EA* An important limitation of the concept of energy availability is that it does not consider energy expenditure from normal daily activity that is not formal exercise, i.e., NEAT (Levine 2004). In relation to the importance of NEAT for energy availability, we must consider three key factors. First, from a purely physiological perspective, there is no difference whether energy is expended during exercise, a subset of physical activity with the objective of improving or maintaining fitness, or other types of physical activity, which is defined as any bodily movement that results in energy expenditure (Caspersen et al. 1985). Therefore, all activity expenditure should be accounted for when determining a ‘true’ energy availability value, though we acknowledge that the assessment of NEAT is technically difficult in the majority of EA assessment scenarios. Second, since NEAT may vary in response to changes in energy balance (Levine et al. 1999; Müller and Bosy-Westphal 2013), it is also likely to be affected by EA. However, the relationship between EA and NEAT has not been studied to the best of our knowledge. Third, NEAT is highly variable between individuals (Levine 2004; Villablanca et al. 2015). While the first two factors are important mainly in controlled trials with crossover design and observational studies, the last issue is particularly important when translating findings of laboratory studies to the field and when considering cross-sectional studies. While it is appreciated that the contribution of NEAT to the total energy budget is proportionally smaller in athletes with a high training load, both because of the high exercise energy expenditure and because of less time available for NEAT, it should not be disregarded as a contributing factor.

In conclusion, until proven otherwise, the lack of consideration of NEAT outside the laboratory in research focusing on EA provides simplicity on the side of calculating EA, but poses it as a potential ‘noise’ factor for the comparison of EA between studies or for using universal thresholds/cut-off values of EA under which metabolic and behavioural adaptations may occur.

**Fig. 2:**
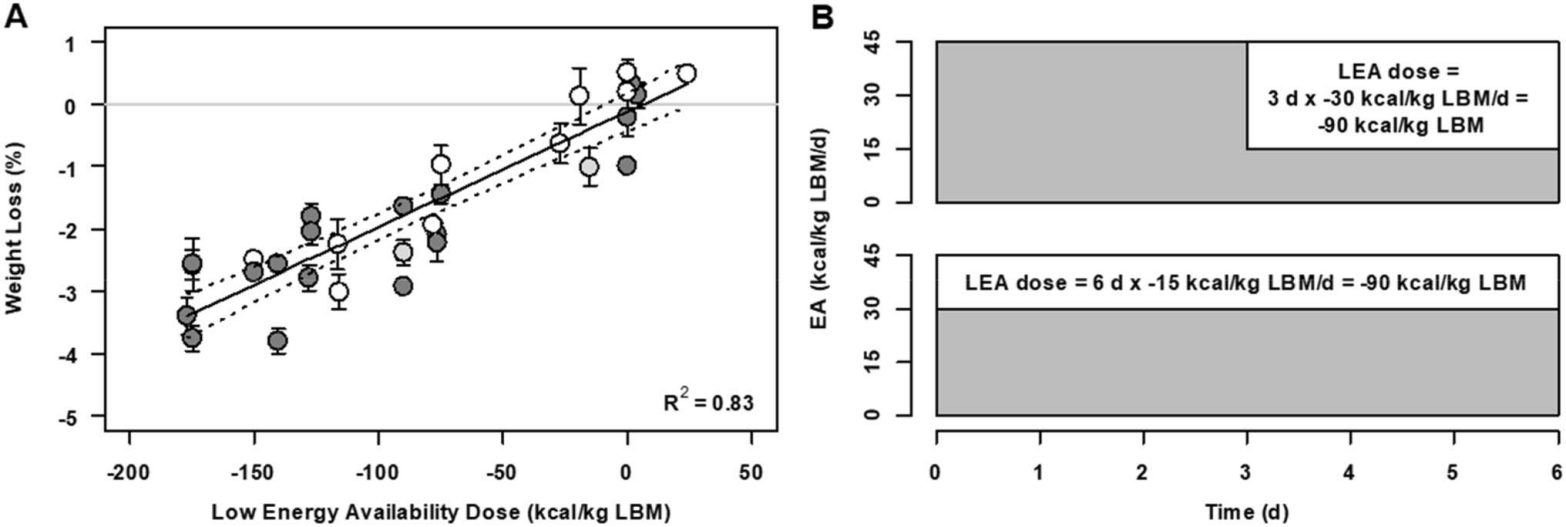
Relationship between energy availability (EA) and changes in body weight after 3–5 days (**a**) using the concept of low energy availability dose’ summarising data from studies manipulating energy availability and measuring weight pre-post intervention (Areta et al. 2014; Ihle and Loucks 2004; Koehler et al. 2016b; Kojima et al. 2020; Loucks 2006; Loucks and Heath 1994b; Loucks and Thuma 2003; Loucks and Verdun 1998; Loucks et al. 1998; Murphy and Koehler 2020; Papageorgiou et al. 2017, 2018). The shading of the circles is representative of the female ratio in each study -dark grey means only women and white only men; the error bars reflect the SEM of the weight loss (if available). Low energy availability dose defined as the total amount of EA under 45 kcal/kg LBM/day. There is a strong correlation between low energy availability dose and decrease in body weight. **b** Exemplifies two different ways of obtaining − 90 kcal/kg LBM low energy availability dose: with 3 days of 15 kcal/kg LBM/day EA (3 days × − 30 kcal/kg LBM/day) or 6 days of 30 kcal/kg LBM/day EA (6 days × − 15 kcal/kg LBM/day)

Another important limitation of the research on EA is that, to date, prospective trials inducing LEA are conceptually very similar to energy deficit interventions, as evidenced by a strong linear relationship between EA and weight loss, at least during the first 3–5 days of reduced energy availability, the most common intervention length in controlled EA trials (Fig. 2). The rapid and drastic body weight change in these scenarios may be partially attributed to a reduction in skeletal muscle glycogen due to reduced carbohydrate availability (Areta and Hopkins 2018; Ishibashi et al. 2020; Kojima et al. 2020) and the water bound to it (Olsson and Saltin 1970; Sherman et al. 1982). The linear association between EA and weight-loss suggests that adaptive mechanisms are not yet evident through body weight changes as a consequence of short LEA interventions.

## Endocrine, metabolic and physiological effects of low energy availability in humans in controlled settings

**Fig. 3:**
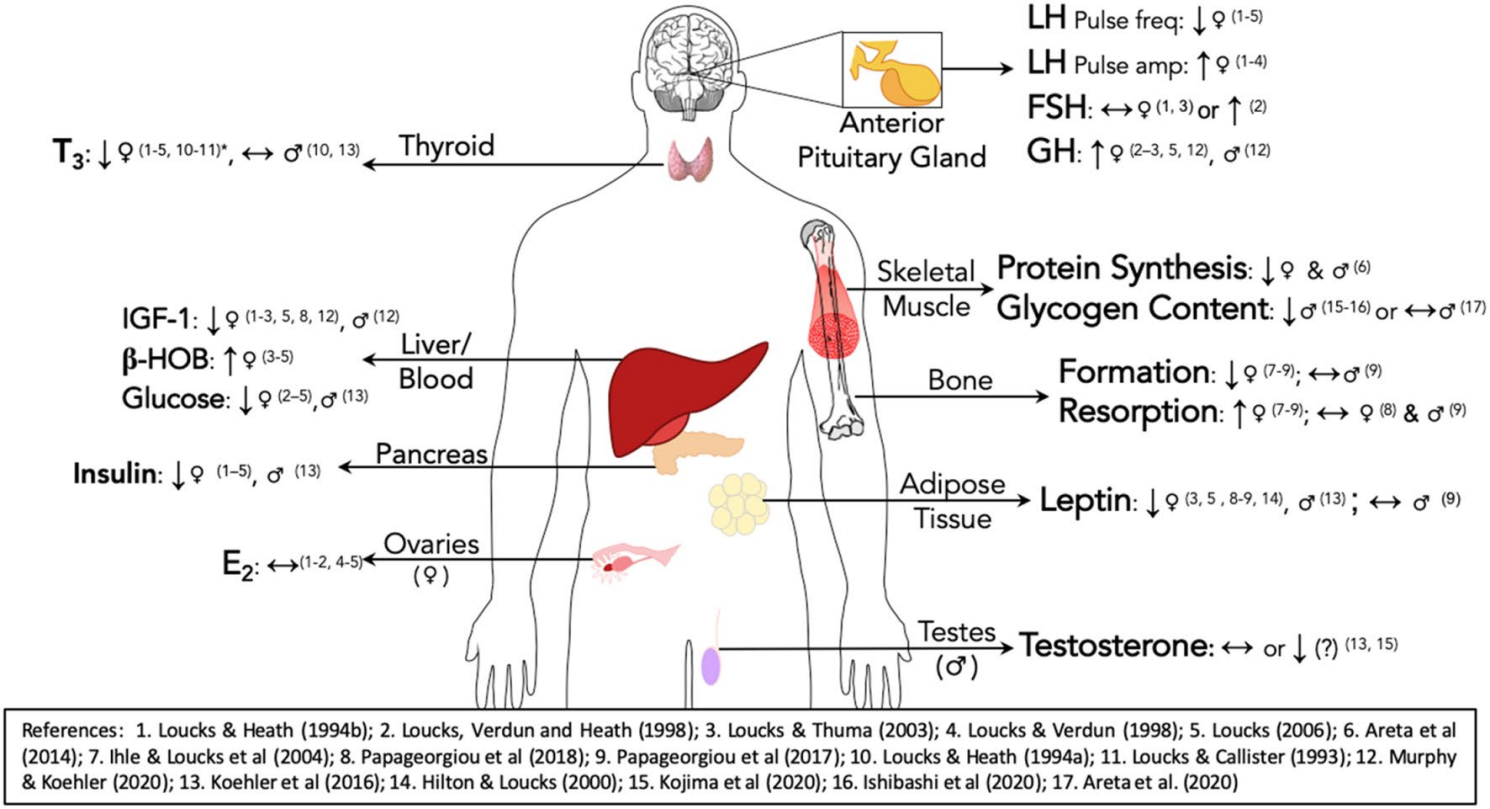
Graphical summary of the effects of short-term (3–5 days) low energy availability (EA) on hormones, blood-borne substrates and skeletal muscle as evidenced in prospective studies. Low energy availability is a powerful stressor that triggers marked hormonal and metabolic responses. Down-regulation of key energy homeostasis-related adipokine leptin may lay upstream of and partially modulate the hypothalamic-pituitary-thyroid, hypothalamic-pituitary–gonadal and GH-IGF-1 axes. LEA also modulates markers of bone formation (decrease), resorption (increase), substrate availability and reduces skeletal muscle protein synthesis. Different tissues/systems are affected in different ways, and this response seems to vary between males and females, potentially due to divergent susceptibility to different levels of EA in different tissues and genders. Male or female gender symbol specifies that research supporting the direction of change (or lack thereof) has been conducted or reported on that gender specifically. *β-HOB* Beta hydroxybutyrate, *FSH* follicle stimulating hormone, *GH* growth hormone, *IGF-1* insulin-like growth-factor 1, *LH* luteinizing hormone, *T*_*3*_ triiodothyronine. Bone formation markers refers to osteocalcin, carboxy-terminal propeptide of type 1 procollagen (P1CP) and N-terminal propeptide of type 1 procollagen (P1NP). Bone resorption markers refers to C-terminal telopeptide of type 1 collagen (β-CTX) and aminoterminal telopeptide of type 1 collagen (NTx)

The initial work on EA was set to unravel the previously unexplained observations of altered hypothalamic-pituitary-ovarian and hypothalamic–pituitary–adrenal axes in female amenorrhoeic athletes in comparison to their eumenorrheic counterparts and sedentary eumenorrheic women (Loucks et al. 1989, 1992). It was unclear if it was an ‘energy drain’ or the stress of exercise that triggered this response. An outstanding body of work led by Prof. Anne Loucks encompassed several rigorously executed clinical trials and established that energy availability, and not stress of exercise, was the underlying cause of these and other endocrine dysregulations in females (Tables 2, 3). Further work by us (Areta et al. 2014, 2020; Koehler et al. 2016b; Murphy and Koehler 2020; Smiles et al. 2015) and others (Ishibashi et al. 2020; Kojima et al. 2020; Papageorgiou et al. 2017, 2018) expanded this area to other physiological systems of interest in both sexes. The current section summarises this research grouped in endocrine systems and tissues, organised hierarchically in (1) General endocrine response (leptin, hypothalamic-pituitary-thyroid axis, and Growth hormone-Insulin-like growth-factor axis and cortisol) (Table 2); (2) hypothalamic-pituitary–gonadal axis (Table 3); (3) Blood-borne metabolic substrates, (4) bone metabolism (Table 4), and, (5) skeletal muscle responses (Table 5), all of which is summarised in a figure (Fig. 3).

### General endocrine response

#### Leptin

Leptin is a key hormone for energy homeostasis. It is secreted by adipocytes and it regulates energy expenditure and intake through hypothalamic control and peripheral tissue metabolism exerting an important top-down regulation of the neuroendocrine axes (Blüher and Mantzoros, 2009; Margetic et al. 2002). In humans, leptin exerts a modulatory effect mainly when its circulating levels decrease, rather than when it circulates above the levels in a normal energy balance state (Ravussin et al. 2014). Despite leptin’s secretion from adipose tissue and its close correlation with fat mass (Considine et al. 1996), a fall in circulating leptin occurs early during food deprivation, well before there are measurable changes in fat mass (Kolaczynski et al. 1996; Patel et al. 2019).

Even though dietary restriction, fasting and exercise have been independently related to decreases in circulating leptin (Fedewa et al. 2018; Voss et al. 2016), evidence suggests that energy status and therefore EA is likely the common denominator. The vast majority of studies measuring leptin after LEA (Table 2) report a decrease with values of ≤ 30 kcal/kg LBM/day, regardless of whether LEA is achieved through dietary restriction alone or in combination with exercise (Hilton and Loucks 2000; Koehler et al. 2016b). Interestingly, LEA not only reduces mean 24 h leptin values but also its daily amplitude (Hilton and Loucks 2000) and it appears to follow a dose–response, with decreasing EA levels resulting in lower circulating leptin values, at least evidenced from the morning fasting values (Loucks and Thuma 2003). To date, only one study has reported similar reductions in leptin in response to LEA (15 kcal/kg LBM/day) in males (Koehler et al. 2016b), whereas another study reported differences in females but not males, suggesting that this may be a parameter where females are more vulnerable to LEA (Papageorgiou et al. 2017).

Despite potential sex differences in the sensitivity of leptin to LEA, overall evidence from the majority of short-term studies is in consonance with the reductions in leptin reported in numerous cross-sectional and observational studies (Elliott-Sale et al. 2018).

#### Hypothalamic-pituitary-thyroid axis

The hypothalamic-pituitary-thyroid axis plays a critical role in the regulation of energy expenditure and adaptive thermogenesis (Kim 2008; McAninch and Bianco 2014) and is likely at least partially mediated by leptin (Blüher and Mantzoros 2009). Thyroid hormones influence key metabolic pathways related to the control of energy balance, and regulate metabolism through acting in the brain, white fat, brown fat, skeletal muscle and pancreas (Mullur et al. 2014) likely through a profound effect on mitochondrial metabolism (Lanni et al. 2016). Low 3,5,3-Triiodothyronine (T_3_) was first observed in amenorrhoeic athletes (Loucks and Callister 1993) and therefore thought likely responsive to EA. The first study on the endocrine effect of EA in humans determined that EA and not the stress of exercise determined thyroid hormone concentrations (Loucks and Callister 1993). This study demonstrated that exercise (performed at 40% and 70% maximal aerobic capacity) did not affect thyroid hormones when EA was maintained at ~ 38 kcal/kg LBM/day (originally reported as kcal/kg BM/day). However, reducing EA to ~ 11 kcal/kg LBM/day for 4 days decreased free and total T_3_, reverse T_3_, and increased thyroxine (T_4_) regardless of performing exercise or not (Loucks and Callister 1993). A subsequent study determined a threshold of ~ 25 kcal/kg LBM/day in females, under which, thyroid hormone dysregulation is evident (Loucks and Heath 1994a). The majority of subsequent studies have reported that T_3_, the active form of the hormone, is consistently reduced in response to LEA at least in females (Table 3), while changes in T_4_ -its precursor- are ambiguous (Loucks and Callister 1993; Loucks and Heath 1994a). In males however, short-term reductions in EA to 15 kcal/kg LBM/day through dietary restriction with or without exercise did not significantly impact T_3_ (Koehler et al. 2016b; Papageorgiou et al. 2017). The clear causal response to short-term exposure to LEA, at least in females, is consistent with prolonged observational studies (Elliott-Sale et al. 2018; VanHeest et al. 2014), making T_3_ a reliable parameter of LEA that shows clear agreement between controlled and cross-sectional studies. The short- and long-term effects on males are unclear, however.

#### Growth hormone-insulin-like growth factor-1 axis and cortisol

Another group of hormones which have consistently been assessed in response to LEA involve those with anabolic and catabolic responses. While in energy balance, pituitary release of growth hormone (GH) stimulates the release of insulin-like growth factor 1 (IGF-1) from the liver, which exerts effects in most tissues and decreases pituitary GH release through a negative feedback loop. During starvation, the liver becomes insensitive to GH, which is compensated by increased pituitary GH release with decreased circulating IGF-1 (Fazeli and Klibanski 2014). Similarly, LEA consistently increases circulating GH with a concomitant reduction of circulating IGF-1 when EA < 20 kcal/kg LBM/day (Table 2). This failure to stimulate hepatic IGF-1 secretion has not only been observed at rest, but also in response to exercise training. For example, a recent study from our laboratory demonstrated that the post-exercise GH/IGF-1 response is disrupted in individuals exposed to LEA. After 2 days of dietary restriction to achieve an LEA of 15 kcal/kg LBM/day, resistance-trained individuals conducted a bout of controlled resistance exercise. In response to LEA, post-exercise IGF-1 area under the curve was reduced by 20–30%, while the exercise-induced GH spike was ~ 2.5 times greater, providing further evidence for an impaired hepatic IGF-1 response in the LEA state (Murphy and Koehler 2020).

In agreement with an impaired GH/IGF-1 axis indicating a decrease in the anabolic response, cortisol, associated to a catabolic response is reportedly increased. Cortisol has not been reported consistently in the LEA literature, but measured as 24 h transverse mean it has been shown to increase with EA ≤ 30 kcal/kg LBM/day in studies incorporating exercise to achieve LEA. However, dietary restriction alone does not seem to be sufficient to elevate circulating cortisol (Table 2).

## Hypothalamic-pituitary–gonadal axis

A series of early studies detailed the effect of EA during the early/mid-follicular phase on circadian variation in key hormones of the hypothalamic-pituitary-ovarian axis through 24 h serial blood sampling (Table 3). The first study in this series in young eumenorrheic sedentary participants determined that 4 days of a reduction of EA to 10 kcal/kg LBM/day through dietary means (without exercise) during the early-mid follicular phase altered the circadian secretion of luteinizing hormone (LH) release. Normal LH pulse frequency was reduced and its amplitude increased, resembling that of females with hypothalamic amenorrhea (Loucks and Heath 1994b). Using the same experimental model but incorporating exercise energy expenditure of 30 kcal/kg LBM/day to achieve similar levels of LEA (~ 13 kcal/kg LBM/day using a different methodology for calculation, see Table 1), showed an alteration of LH pulse frequency and amplitude comparable to that achieved by restricted energy intake only, indicating the predominant effect of LEA rather than an exercise effect on the hypothalamic-pituitary-ovarian axis (Loucks et al. 1998). Subsequently, a study exposing the same population to LEA of 10 or 20 or 30 kcal/kg LBM/day EA, through dietary restriction and exercise energy expenditure of 15 kcal/kg LBM/day for 5 days, confirmed that LH pulsatility decreased below a threshold of 30 kcal/kg LBM/day EA (that is at EA levels of 10 and 20 kcal/kg LBM/day), when compared to the control trial at 45 kcal/kg LBM/day (Loucks and Thuma 2003). Despite the clear effect of EA on LH pulsatility, these studies also demonstrated that other reproductive outcomes, namely follicle stimulating hormone (FSH), remained mostly undisturbed, and mean 24-h estrogen (E_2_) exposure was decreased slightly only at the most severe LEA levels of 10 kcal/kg LBM/day (Table 2). Given that hypoestrogenism is an important link between menstrual health and bone and cardiovascular health in the *triad* (De Souza et al. 2014) and RED-S models (Elliott-Sale et al. 2018), these findings suggest the suppressive impact of LEA on E_2_ requires more time to develop when EA levels are > 10 kcal/kg LBM/day, but such causal evidence is currently lacking.

Interestingly, a retrospective analysis of menstrual function and hormonal responses during three consecutive menstrual cycles showed that previously sedentary women demonstrated a decrease in estrogen regardless of EA levels (low, moderate and high), which were achieved with increased exercise energy expenditure in all individuals and varying dietary restriction. This observation suggests that increasing exercise energy expenditure per se rather than reducing EA may have suppressed this and other sex hormones (Lieberman et al. 2018; Williams et al. 2015). Additionally, though there was an increase in the likelihood of menstrual disturbances with decreasing EA during this period, a threshold of 30 kcal/kg LBM/day EA did not guarantee menstrual disturbances (or the lack thereof) with this timeline (Lieberman et al. 2018). A potential factor influencing in a woman’s susceptibility to LEA is gynaecological age, as shown by the findings that women of younger gynaecological age are more susceptible to incidences of anovulation and short luteal phases (Loucks, 2006). This study reported that 5 days of reducing EA to 10 through exercise (15 kcal/kg LBM/day) and dietary restriction reduced LH pulse frequency in adolescents but not in adults (Loucks 2006).

In males, the research investigating the effect of LEA on the hypothalamic-pituitary–gonadal axis is limited. Even though exercising males at risk of energy deficiency may show signs of hypogonadism (Hackney 2020) and impaired reproductive capacity (De Souza et al. 1994), the response of testosterone to LEA has only been investigated in two studies. Our research has shown that 4 days LEA at 15 kcal/kg LBM/day had no effect on resting testosterone levels in comparison to normal energy availability (NEA) (Koehler et al. 2016b), but a recent report shows a decrease in resting testosterone within a group of athletes after 3 days of EA of 19 kcal/kg LBM/day (Kojima et al. 2020). Altogether these findings suggest that even though reduced testosterone and hypogonadism are a possible consequence of prolonged LEA, there is currently insufficient evidence to establish a dose–response relationship or time course between LEA and low testosterone, nor whether exercise per se or LEA are the primary driver of reductions in circulating testosterone.

### Blood-borne metabolic substrates (glucose, β-hydroxybutyrate and free fatty acids) and insulin

Though metabolic substrates and insulin may not necessarily be modulators of the effect of LEA, but a product of substrate deprivation, they have a central role in metabolism and physiology making it pertinent to analyse their responses in different prospective studies.

Resting blood glucose and insulin were consistently shown to be reduced in the vast majority of the studies in response to EA ≤ 30 kcal/kg LBM/day (both in morning values and 24 h transverse mean), with further reductions in EA resulting in more pronounced responses (Table 2). Accordingly, morning blood concentration of the ketone body β-Hydroxybutyrate (β-HOB), have consistently been shown to be elevated in response to LEA in a step-wise manner (Table 2). The majority of the studies have not measured free fatty acids and glycerol as markers of increased lipolytic activity, but in consonance with the glucose, insulin and β-HOB responses, we have shown increases in fasting concentrations as an effect of LEA in males (Koehler et al. 2016b).

The reduced availability of carbohydrates with LEA is, accordingly, reflected in increased rates of fat oxidation during submaximal exercise. Our research has shown that fat oxidation is increased during submaximal exercise after 5-days of LEA of 15 kcal/kg LBM/day, induced by dietary restriction and exercise (Murphy et al. 2018), and also after short (< 24 h) exposure to LEA of ~ 20 kcal/kg LBM/day vs. 45 kcal/kg LBM/day with carbohydrate-matched diets (Areta et al. 2020). Taken together, these data support the idea that LEA also induces low-carbohydrate availability, posing it as a potential key parameter in relation to LEA as previously suggested (Loucks and Thuma, 2003).

### Bone metabolism

Considering the association between reduced bone mineral density, osteoporosis and stress fracture prevalence in populations at risk of chronic LEA (De Souza et al. 2014), bone metabolism has been a topic of focus in research (Table 4). Bone remodelling is a slow process that occurs continually through resorption and formation of its matrix (Dolan et al. 2019) and detecting changes in bone mineral density using imaging techniques may take months or years (Dolan et al. 2019; Villareal et al. 2016). Studies investigating short-term effects of LEA in controlled settings have therefore focused on evaluating changes in blood-borne markers of bone formation and resorption, which can be predictors of long-term changes in bone mineral density (Dolan et al. 2019; Villareal et al. 2016).

Decreasing EA from 45 (control) to 30, 20 or 10 kcal/kg LBM/day for five days in females, first showed markers of bone formation to be more sensitive to LEA than markers of bone resorption (Ihle and Loucks 2004). Bone formation markers such as serum type I procollagen carboxy-terminal propeptide (*P1CP*) and osteocalcin (*OC*) were reduced in every LEA condition, with P1CP showing a linear step-wise decrease between 45 and 10 kcal/kg LBM/day EA. Similarly, OC was reduced in every LEA condition but plateaued at 20 kcal/kg LBM/day EA. Instead, the bone resorption marker N-terminal telopeptide of type 1 collagen (*NTx*) concentrations only increased at more extreme LEA levels of 10 kcal/kg LBM/day. Provided this research included daily exercise in all conditions and provided exercise modulates bone metabolism, it remained unclear how exercise impacted the bone response during LEA.

Papageorgiou et al. (2018) investigated the effect of LEA of 15 kcal/kg LBM/day for 3-days in bone metabolism induced through dietary-restriction or exercise energy-expenditure alone. This study showed a decrease in bone formation marker procollagen type 1 N-terminal propeptide (P1NP) regardless of whether LEA was induced by exercise or dietary restriction, compared to the control condition. Similarly to Ihle and Loucks (2004), this level of energy availability did not affect another marker of bone resorption, β-carboxyl-terminal cross-linked telopeptide of type I collagen (β-CTX), in either LEA state compared to control (Papageorgiou et al. 2018), supporting previous findings that bone formation may be more sensitive to LEA than resorption.

However, these findings may only be applicable to females, given that males seem to be more resilient in general to the bone effects of LEA. For example, Papageorgiou et al. (2017) assessed response to 5 days of LEA (15 kcal/kg LBM/day) in women and men, all while incorporating daily aerobic exercise. Female participants exhibited a reduction in P1NP and an increase in β-CTX following LEA, but no changes were apparent for males. Similarly, we have shown no effect of three days of LEA (15 kcal/kg LBM/day) upon P1NP concentration in a cohort of five males and two females (Murphy and Koehler 2020).

In conclusion, current evidence shows that LEA results in greater suppression of markers of bone formation than increased markers of bone resorption and this effect has been documented in females but not in males. However, bone resorption and formation, are not independent processes—as evidenced by the bone turnover regulation through the receptor activator of nuclear factor-κB ligand/osteoprotegerin pathway (Boyce and Xing, 2007)—, and mechanistic details regarding the modulatory effect of EA should be unravelled by future research. Importantly, further experimental data is required to determine which LEA levels affects male bone markers. Though stress fractures seem to be approximately three times more frequent in exercising females than in males (Edouard et al. 2015; Wentz et al. 2011), cross-sectional data from male athletes susceptible to LEA suggests that the prevalence of markers of impaired bone health is also problematic among male athlete populations (Tenforde et al. 2016).

### Skeletal muscle and physical performance

Despite the prominent role of skeletal muscle on health and physical performance, the effect of LEA on skeletal muscle physiology and performance has received surprisingly less attention than other systems (Table 5). This subsection reviews the effect of LEA on muscle protein synthesis, autophagy, markers of response to endurance-type training and aerobic performance.

To date, the most prominent effect of LEA on skeletal muscle is probably the decrease of myofibrillar protein synthesis (MPS) (Areta et al. 2014). Our research showed that EA of 30 kcal/kg LBM/day for 5 days, reduced resting MPS by ~ 27% (Areta et al. 2014). Our findings also showed that sex had no impact on the effect of LEA on MPS (Areta et al. 2014). These findings agree with parallel impairment of the anabolic hormone milieu (Table 2), as described above.

Despite the down-regulation of MPS, upstream intracellular signalling and gene expression were largely unaffected by LEA (Areta et al. 2014; Smiles et al. 2015). LEA had no effect on signalling of the mTOR or AMPK pathways or mRNA expression of ubiquitin ligases (MuRF-1 and Atrogin), but reduced the expression of system L amino-acid transporter SLC7A5 mRNA, an important transporter of leucine into skeletal muscle (Table 4). Further research on the same sample set showed mostly no effect on autophagy signalling, only reducing the autophagy-related gene protein 5 (cAtg5) content, suggesting that autophagy is not a substantial contributor to proteolysis during early LEA (Smiles et al. 2015). This study also showed that mRNA of markers of mitochondrial biogenesis such as PGC-1α and Sirt1 were largely unaffected by LEA (Smiles et al. 2015).

Similarly, a recent study from our laboratory shows that short (< 24 h) exposure to LEA of ~ 20 kcal/kg LBM/day did not negatively affect early markers of skeletal muscle adaptation to aerobic-type training. We observed no differences in skeletal muscle glycogen content with carbohydrate-matched diets after exercise, no impaired response on mitochondrial biogenesis-related genes or down-regulated AMPK signalling pathway early (+ 3.5 h) during recovery (Areta et al. 2020). This suggests that acute and short-term exposure to LEA may not impair oxidative adaptive response in skeletal muscle, and also possibly does not affect aerobic capacity either.

Indeed, a recent study addressing the effect of EA on skeletal muscle glycogen and performance in well-trained runners showed that 3 days of EA of 19 kcal/kg LBM/day, had no effect on exercise capacity on a time-to-fatigue test at 90% VO_2max_ lasting ~ 20 min duration, (Kojima et al. 2020). The reduction of muscle glycogen was reduced from day 1 of the intervention, an observation that was replicated in a subsequent study with a similar experimental design (Ishibashi et al. 2020). This is likely due to the combination of high training load and low-carbohydrate availability, rather than energy availability itself (Areta and Hopkins 2018; Bergström et al. 1967; Olsson and Saltin 1970). Continuous long-term LEA may, however, negatively affect endurance performance or adaptation to training (VanHeest et al. 2014). However, to date, there has been no long-term intervention studies that control LEA and assess performance.

In conclusion, these early findings seem to support the idea that while short-term LEA reduces muscle protein synthesis, adaptations towards an oxidative phenotype on skeletal muscle and aerobic capacity are not impaired. This assumption is also supported by field observations of endurance athletes with signs of acute and chronic low energy availability showing high capacity of aerobic performance (Areta et al. 2020; Fudge et al. 2006; Stellingwerff 2018) as well as comparable maximal aerobic capacity between normal athletes and those showing signs of chronic LEA (Loucks et al. 1989; Melin et al. 2015). From an evolutionary perspective the response to LEA could be interpreted as an organism maintaining functions that are essential for survival and food procurement (locomotion), while reducing allocation of energy and resources for tissues and systems that are not immediately essential for survival (e.g. bone metabolism and reproductive function).

## Perspectives and future research

Low energy availability shows to be a powerful physiological stressor that produces a dramatic shift in the endocrine milieu and metabolic response within days (Fig. 3). However, our current understanding on the dose, timeline and tissue-specific effects on different populations is rather limited. Due to the potential health consequences of chronic insufficient dietary energy with concomitant physical activity, this concept is relevant not only for athletes but also for the general population seeking weight-management. A large body of research is waiting to be performed to develop our understanding of the physiological effects of LEA, and therefore allow us to develop ways in which its negative effects can be minimised or eliminated. To do so, we must recognise the gaps and limitations of our current knowledge and practice.

*Prospective studies are short in duration* The first point that stands out in current research findings of LEA is the short duration of experimental studies of 3–5 days (Tables 2, 3, 4 and 5), likely representing early endocrine perturbations only. Strict control of exercise, dietary intake and sample collection is resource-intensive for researchers and participants. For the latter, normal daily activities need to be altered or sacrificed to fit with experimental protocol demands, which makes it extremely difficult to maintain for longer periods. Short interventions, however, allow documentation of early endocrine responses to LEA, as well as to test how different interventions may minimise its negative effects (Areta et al. 2014; Murphy and Koehler 2020). We believe that longer interventions will allow researchers to determine the causality between LEA and a series of responses that remain hypothetical such as, a decoupling between LEA and weight loss (Fig. 2), endocrine dysregulations and functional outcomes such as physical capacity. While numerous discoveries await to be unravelled from short-term studies, future studies could also use a ‘blended’ approach, incorporating regular laboratory-based screening and allowing participants in free-living conditions following rigorous control of energy expenditure and intake. From an ethical perspective, however, it is important that the severity and duration of future prospective studies should be such that it allows for novel discoveries, but not excessive so that it instigates potential irreversible consequences of chronic LEA, such as significant reduction of BMD.

*Low energy availability load* One important question that these type of longer term ‘blended’ studies would allow researchers to answer is whether there is a cumulative dose of LEA which elicits an endocrine response. It is currently unclear whether longer but less severe reductions in EA elicit the same responses as the short-term interventions with very low LEA conditions predominantly employed in controlled experiments. Such an approach could identify an ‘LEA load’ that an individual can face prior to experiencing a meaningful degree of endocrine perturbation and metabolic adaptation, or confirm the existence of LEA thresholds independent of duration. As with an exercise stressor (‘training-load’), correct periodisation of energy availability would allow the optimisation of weight-loss and body composition while maintaining health and physical capacity (Areta et al. 2020; Stellingwerff 2018). Therefore, here we propose the concept of ‘*low energy availability load*’ (Fig. 2) for laboratory-based studies, defined as the cumulative amount of energy availability under what is typically considered adequate (45 kcal/kg LBM). For example, from a mathematical standpoint, 3 days of EA of 15 kcal/kg LBM/day are equivalent to 6 days of EA of 30 kcal/kg LBM/day (LEA load: 90 kcal/kg LBM). Figure 2, shows the relationship between calculated LEA load and the level of weight-loss (considered as a marker of energy stress) reported in controlled energy availability interventions. This theoretical concept will have to be tested with rigorous science before it can be used widely, but we think it could have important implications for assessing EA thresholds and the time and dose that is required to elicit endocrine and metabolic responses of LEA.

*There is limited research on males* Due to the early observations of high prevalence of menstrual dysfunction and low bone mineral density in exercising females, the majority of research has been carried out in females. This topic is therefore probably one of the few in exercise nutrition that has been investigated more in females than in males. The effects of LEA on males remains largely uncharacterised, although from research on markers of bone metabolism (Papageorgiou et al. 2017) (Table 4) and endocrine response (Koehler et al. 2016b) (Table 2) it appears that men’s physiology is more resilient to LEA. We can speculate that the differences in the endocrine and physiological response between sexes may be associated to the energetic demands of maintaining reproductive system and gestation, which are significantly higher for females than males (Bronson 1985). As such, female physiology may be more sensitive to reductions in EA to ensure successful gestation in periods of reduced or low EA. In this context, reduction in leptin in females at a higher threshold may represent an early signal for energy conservation, whereas a reduction in bone metabolism may represent also a re-allocation of energy for other essential systems to ensure survival and reproductive success. However, other systems such as reduction of skeletal muscle protein synthesis respond comparably in both sexes (Areta et al. 2014) (Table 5). Future work should first determine if short periods of LEA can disrupt male endocrine systems as in females and replicate the landmark studies of Prof. Loucks to determine what levels of energy availability are likely to affect male physiology.

*Caution must be exercised when applying lab-based research findings on the field* In line with the previous points, it is important to call for practitioners and researchers to be cautious when extrapolating laboratory-based thresholds established in short-duration, well-controlled studies for interpreting EA levels observed in the field with athletes and general population both in longitudinal and cross-sectional analyses. There are several factors discussed in this review that make the translation difficult and are enumerated here: (1) EA assessment in the field is challenging and prone to errors, (2) The way in which EA has been calculated in different studies is different, and there does not seem to be consensus on how it should be calculated in the field, (3) The extent to which NEAT determines EA is unknown, especially when there is a high variation in daily NEAT, (4) The early responses to LEA may change when LEA is sustained over prolonged periods, (5) The population in which field studies are carried out is much more heterogeneous than lab-based studies which have been conducted primarily in young sedentary women, (6) the ecological validity of LEA studies in relation to EA daily variability and macronutrient composition is unknown.

*LEA in laboratory-based studies is artificially homogenous* In relation to the last point, virtually all controlled LEA experiments have employed constant, homogenous daily LEA levels. In contrast, alternating days of very low LEA and normal EA would represent more ecologically valid studies. As observed on the field with endurance athletes (Areta JL, *manuscript in preparation*) and general population (Bray et al. 2008; Champagne et al. 2013), individuals in free-living conditions demonstrate considerable day-to-day variation in energy intake and exercise energy expenditure.

*The timeline of recovery from periods of LEA is unknown* Along the previous concept that daily EA is not homogenous, it is unclear if periods of energy ‘supercompensation’ involving an EA ‘surplus’ can rescue the effects of short or long-term LEA. It appears that the recovery of suppressions of the hypothalamic-pituitary–gonadal axis observed after periods of LEA happens within days upon refeeding as observed in field studies inducing drastic energy deficits in male soldiers (Friedl et al. 2000). To address if super-compensating EA after a short period of LEA would restore normal endocrine function in females, Loucks et al. (1998) exposed young females to 5 days of an EA of 10 kcal/kg LBM/day followed by an aggressive one-day refeeding to achieve 77 kcal/kg LBM/day EA. Findings revealed that this aggressive refeeding regimen failed to restore T_3_ levels and only partially restored LH pulsatility (Loucks and Verdun 1998). This study suggests that in humans, opposite to other mammalian species, restoration of the normal endocrine milieu after resuming adequate energy availability is a slow process, something that is supported by our (Areta et al. 2020), and others’ (Łagowska et al. 2014; Mallinson et al. 2013; Stickler et al. 2019) observations of periods of weeks and months to resume normal menstrual cycle after prolonged periods of amenorrhea and oligomenorrhea.

*The effect of dietary composition during LEA is largely unknown* It is also not clear whether availability of specific macronutrients in particular plays a more prominent role in development metabolic adaptation or if EA itself, regardless of dietary macronutrient composition, is the key parameter. The majority of EA studies provide dietary control with a set proportion of macronutrients, that are reduced by the same amount in the LEA intervention (Tables 2, 3, 4 and 5). Our work has shown that protein intake overlayed on top of resistance training can potentially be a treatment to minimise at least some of the negative consequences on acute skeletal muscle protein synthesis response (Areta et al. 2014). However, this does not seem to have an effect on the acute post-training GH/IGF-1 response (Murphy and Koehler 2020). Recent work from our group (Hammond et al. 2019) and others (Heikura et al. 2020) comparing low-carbohydrate/high-fat diets with high carbohydrate/low-fat diets suggest that carbohydrate availability rather than energy availability may be a key factor in modulating the osteoclastic/osteogenic bone response. This parallels speculations on brain requirements of glucose dictating a central neuroendocrine response, whereby glucose availability of less than ~ 130 g/day results in perturbation of the endocrine milieu (Loucks and Thuma 2003). Future studies should address the effect of contrasting macronutrient content on periods of LEA to determine the modulating role of each macronutrient.

*There are no experimental studies showing a direct link between LEA and RMR suppression or uncoupling with weight-loss* Despite prolonged energy deficits being linked to adaptive reductions in RMR (Müller and Bosy-Westphal 2013; Müller et al. 2015) and RMR suppression being considered a hallmark of chronic LEA (Loucks et al. 2011), to date there has only been one study that has assessed this in experimental settings. Kojima et al (2020) showed that 3 days of EA of 19 kcal/kg LBM/day had no effect on RMR. Moreover, the hypothesised downstream effect of this purported energy conservation—uncoupling between LEA and weight loss—has not been shown directly in experimental settings. In short-term prospective studies there is no uncoupling between LEA and weight loss (Fig. 2a). Unless we can show experimentally that the rate of weight loss decreases as a set level of LEA is maintained over a prolonged experimental intervention, to date there is insufficient evidence to support the hypothesis that EA is fundamentally different to energy balance in relation to changes in body weight.

*It is unclear to what extent endogenous energy stores interact with energy availability* The majority of the research has been carried in individuals with relatively homogenous body composition, i.e., neither extremely lean nor equipped with large fat reservoirs. As discussed in Sect. “The birth and history of energy availability as a concept and its calculation”, the original concept of energy availability referred to oxidable substrate availability and did not differentiate between endogenous and exogenous sources (Schneider and Wade 1989, 1990b). While obese individuals can still show signs of low energy availability after 3 months of extreme food deprivation by developing functional hypothalamic amenorrhea while still obese (Di Carlo et al. 1999), it remains unknown whether more nuanced reductions in energy availability affect individuals with different body compositions differently and if endogenous stores provide some resilience against LEA.

## Conclusion

The concept of energy availability arose from studies on mammalian ecology and was subsequently applied in humans together with the introduction of a simple algebraic calculation, which has evolved over time. This concept is helpful for delineating the effects of energy deprivation from the effects of exercise and focuses on physiological dysregulations rather than its weight-loss effect. While well-controlled trials show that low energy availability results in altered endocrine and physiological responses, what exactly qualifies as low energy availability relative to time of exposure remains unclear, provided the majority of these studies were short in duration (≤ 5 days).

Evidence shows that short-term responses to low energy availability modulate energy homeostasis through leptin and has a modulatory effect on neuro-endocrine axes such as the pituitary-hypothalamic-thyroid, pituitary-hypothalamic-gonadal and GH-IGF-1 axes, as well as down-regulating muscle protein synthesis and bone metabolism. These responses are thought to antecede clinical complications that include, but are not limited to, reproductive function impairment, stress fractures, loss of muscle mass and impaired physical capacity. Given that this concept arose initially from observations of complications in female athletes (amenorrhea and osteoporosis), historically there has been more research on this topic in females. Although current research on males is limited, early findings suggest that men may be less sensitive to reductions in energy availability.

While the concept of energy availability has developed through observations in highly active individuals (athletes), the current obesity epidemic requires the incorporation of low energy availability with concomitant exercise to achieve healthy body weight in large parts of industrialised populations, while minimising its negative effects. Therefore, we believe that the concept can prove useful for informing research relevant to public health. We believe that collaborative research addressing these questions to thoroughly understand the effects of energy and macronutrient availability will allow us to optimise training-nutrient interventions to achieve peak performance in elite athletes as well as guiding practices in the general population to achieve optimal health.

## Abbreviations

AMPK: AMP-activated protein kinase
β-CTX: β-Carboxyl-terminal cross-linked telopeptide of type I collagen
β-HOB: β-Hydroxybutyrate
CIT: Cold-induced thermogenesis
DIT: Dietary-induced thermogenesis
*E*_2_: Estrogen
EA: Energy availability
EEE: Exercise energy expenditure
EI: Energy intake
FSH: Follicle stimulating hormone
GH: Growth hormone
IGF-1: Insulin-like growth factor 1
LBM: Lean body mass
LEA: Low energy availability
LH: Luteinizing hormone
MPS: Myofibrillar protein synthesis
NEAT: Non-exercise activity thermogenesis
*NTx*: N-terminal telopeptide of type 1 collagen
OC: Osteocalcin
P1CP: Type I procollagen carboxy-terminal propeptide
T_3_: 3,5,3-Triiodothronine
T_4_: Thyroxine
RED-S: Relative energy deficiency in sport
RMR: Resting metabolic rate
